# eRegTime—Time Spent on Health Information Management in Primary Health Care Clinics Using a Digital Health Registry Versus Paper-Based Documentation: Cluster-Randomized Controlled Trial

**DOI:** 10.2196/34021

**Published:** 2022-05-13

**Authors:** Mahima Venkateswaran, Zaher Nazzal, Buthaina Ghanem, Reham Khraiwesh, Eatimad Abbas, Khadija Abu Khader, Tamara Awwad, Taghreed Hijaz, Mervett Isbeih, Kjersti Mørkrid, Christopher James Rose, J Frederik Frøen

**Affiliations:** 1 Centre for Intervention Science in Maternal and Child Health University of Bergen Bergen Norway; 2 Global Health Cluster Division for Health Services Norwegian Institute of Public Health Oslo Norway; 3 Faculty of Medicine and Health Sciences An-Najah National University Nablus Occupied Palestinian Territory; 4 Palestinian National Institute of Public Health Al-Bireh Occupied Palestinian Territory

**Keywords:** time-motion study, clinical workflow, digital health intervention, eRegistry, antenatal care, cluster-randomized controlled trial, digital health, child health registry, eRegistry, primary care, health information, primary care

## Abstract

**Background:**

Digital health interventions have been shown to improve data quality and health services in low- and middle-income countries (LMICs). Nonetheless, in LMICs, systematic assessments of time saved with the use of digital tools are rare. We ran a set of cluster-randomized controlled trials as part of the implementation of a digital maternal and child health registry (eRegistry) in the West Bank, Palestine.

**Objective:**

In the eRegTime study, we compared time spent on health information management in clinics that use the eRegistry versus the existing paper-based documentation system.

**Methods:**

Intervention (eRegistry) and control (paper documentation) arms were defined by a stratified random subsample of primary health care clinics from the concurrent eRegQual trial. We used time-motion methodology to collect data on antenatal care service provision. Four observers used handheld tablets to record time-use data during one working day per clinic. We estimated relative time spent on health information management for booking and follow-up visits and on client care using mixed-effects linear regression.

**Results:**

In total, 22 of the 24 included clinics (12 intervention, 10 control) contributed data; no antenatal care visits occurred in the other two clinics during the study period. A total of 123 and 118 consultations of new pregnancy registrations and follow-up antenatal care visits were observed in the intervention and control groups, respectively. Average time spent on health information management for follow-up antenatal care visits in eRegistry clinics was 5.72 minutes versus 8.10 minutes in control clinics (adjusted relative time 0.69, 95% CI 0.60-0.79; *P*<.001), and 15.26 minutes versus 18.91 minutes (adjusted relative time 0.96, 95% CI 0.61-1.50; *P*=.85) for booking visits. The average time spent on documentation, a subcategory of health information management, was 5.50 minutes in eRegistry clinics versus 8.48 minutes in control clinics (adjusted relative time 0.68, 95% CI 0.56-0.83; *P*<.001). While the average time spent on client care was 5.01 minutes in eRegistry clinics versus 4.91 minutes in control clinics, some uncertainty remains, and the CI was consistent with eRegistry clinics using less, the same, or more time on client care compared to those that use paper (adjusted relative time 0.85, 95% CI 0.64-1.13; *P*=.27).

**Conclusions:**

The eRegistry captures digital data at point of care during client consultations and generates automated routine reports based on the clinical data entered. Markedly less time (plausibly a saving of at least 18%) was spent on health information management in eRegistry clinics compared to those that use paper-based documentation. This is likely explained by the fact that the eRegistry requires lesser repetitive documentation work than paper-based systems. Adoption of eRegistry-like systems in comparable settings may save valuable and scarce health care resources.

**Trial Registration:**

ISRCTN registry ISRCTN18008445; https://doi.org/10.1186/ISRCTN18008445

**International Registered Report Identifier (IRRID):**

RR2-10.2196/13653

## Introduction

Digital health interventions (DHIs) have the potential to close critical health system gaps toward achieving universal health coverage in low- and middle-income countries (LMICs) [1]. The 2019 World Health Organization (WHO) guideline on DHIs recommends 9 digital interventions for health system strengthening, given certain context-specific implementation considerations [1]. With the release of the guideline, the WHO highlighted the need for better evidence not only on effectiveness of DHIs but also on other policy-relevant questions such as feasibility, cost-effectiveness, and efficiency [1]. A systematic review summarizing evidence on the time efficiency of electronic health records showed that health workers spent less time on documentation [2], while another review showed an increase in documentation time [3]. Both these reviews were conducted in hospital settings in high-income countries. Such evidence is scarcely applicable in LMICs, where clinical tasks and workflow processes as well as the design and purpose of the DHIs are vastly different. We did not find any systematic reviews of studies of efficiency of DHIs in LMICs.

Despite several demonstrated benefits of using DHIs such as improved service quality and coverage [4], data use and information exchange [5], and health outcomes [6,7], challenges such as increased workloads and stress for care providers have also been reported [1]. In LMICs, client loads are heavy and human resources for health care are scarce [8]. In addition to documenting clinical data, health workers in LMICs typically spend considerable amounts of time on reports of aggregate data to their own Ministries of Health and, in many instances, also to donors and program-based funding agencies [9,10]. While DHIs have the potential to save health workers’ time and thereby improve efficiency of the health system, realizing efficiency goals depends on the implementation strategy. Explicit efforts should be made to reduce health workers’ documentation burden, such as removing duplicate and repetitive documentations on paper [11]. In many health systems in LMICs, health workers using DHIs continue to record the same information both digitally and on paper, resulting in longer times spent in data management activities [1]. Compounded with high client loads, such inefficiencies may adversely affect time spent on patient care.

An eRegistry is a digital health information system consisting of client records for tracking clients longitudinally [12]. In Palestine, a Maternal and Child Health eRegistry has been implemented for antenatal, postnatal, and newborn care services in governmental primary health care clinics. The longitudinal data captured in the eRegistry drive health worker clinical decision support based on national guidelines. Embedded in the implementation was a set of cluster-randomized controlled trials; the eRegQual trial assessed the effectiveness of eRegistry’s clinical decision support versus paper-based client records for improving the quality of antenatal care [13,14].

The aim of the eRegTime study was to evaluate the time spent on health information management in eRegistry clinics compared to clinics performing paper-based documentations.

## Methods

### Study Design, Data Collection, and Outcome Measures

Detailed descriptions of the eRegTime study methodology have been published in the protocol [15]. Briefly, the eRegTime study was conducted in public primary health care clinics providing antenatal care services in the West Bank, in the setting of a cluster-randomized controlled trial (eRegQual), where the eRegistry’s clinical decision support system for antenatal care was evaluated. Of the 119 clusters (primary health care clinics) included in the eRegQual trial, 60 were included in the intervention arm and received the eRegistry with clinical decision support. Built in the District Health Information Software 2 (DHIS2) tracker [16], the eRegistry is accessed through a web-based browser on desktop computers, where care providers enter clinical information in digital client records. The remaining 59 clusters, included in the control arm, continued to use paper-based documentation. The primary health care clinics in the West Bank are staffed by different cadres of health workers including nurses, midwives, and doctors with training in maternal and child health, and obstetricians. The nurse-midwife has the responsibility for most of the documentations in client records and compiling aggregate public health reports for the Ministry of Health, Palestine, and was the only group included for observations in the eRegTime study.

For inclusion in the eRegTime study, two criteria were applied to the primary health care clinics in the eRegQual trial: (1) having only 1 nurse or 1 midwife providing antenatal care services on a given workday (to maintain a 1:1 subject-to-observer ratio) and (2) having, on average, at least 1 booking visit per workday (to capture sufficient antenatal booking visits). A total of 41 clinics were eligible for the time-motion study (20 intervention clusters and 21 control clusters). Of these, 24 clinics (12 eRegistry clinics and 12 paper clinics) were selected by random sampling stratified by laboratory availability, which we reasoned could affect care providers’ activities and clinical workflow. Sampling of clinics was done by researchers independent of the study team. Data on clinic staffing and number of antenatal care visits were derived from an inventory assessment of primary health care clinics in the West Bank, conducted in 2014.

Prior to the eRegTime study, we mapped the workflow in clinics using paper-based documentations (control group), and in clinics using the eRegistry (intervention group) [17]. Our findings showed that a typical workday consisted of client consultations in the mornings, when the nurse or midwife provided routine antenatal care and referred clients to higher levels of care, if appropriate. Clinical documentations were carried out in digital client records in the eRegistry in the intervention group. In the control group, paper-based client records were used. Care providers in intervention and control groups maintained a register book of key indicators for reporting purposes. In addition, in both groups, essential clinical information was documented in a client-held handbook for maternal and child health. Apart from the format (ie, digital vs paper), the eRegistry’s digital client records and the paper-based records contained identical documentation requirements. Afternoon sessions were typically reserved for compiling public health reports, and the nurse or midwife gathered the required information from the client records and registers to calculate aggregate indicators. In the intervention group, automated aggregate reports could be generated in the eRegistry, while in the control group, the reporting was paper-based. Booking visits, when registrations of new pregnancies occur, as well as follow-up antenatal care may be conducted in the primary health care clinics on a given day.

The data collection tool was designed on the basis of the findings from the mapping; a Microsoft Access template from the US Agency for Healthcare Research and Quality [18] was customized for our context. A total of 10 task categories were included in the data collection tool, with each task accompanied by a time stamp. The tool was designed to capture the full range of activities performed by a nurse or midwife in providing antenatal care on a typical workday.

Four data collectors were trained in the use of the data collection tool using the time-motion methodology. Following training, two data collectors individually conducted pilot observations on the same antenatal care consultations in clinics that were not part of the study. That is, 2 observers gathered data on the same day from a clinic using paper-based documentations (control group), while the other 2 observers gathered data from an eRegistry clinic (intervention group). The observers were instructed to only record the primary task in the data collection tool, in case of multitasking by the care provider.

**Figure 1:**
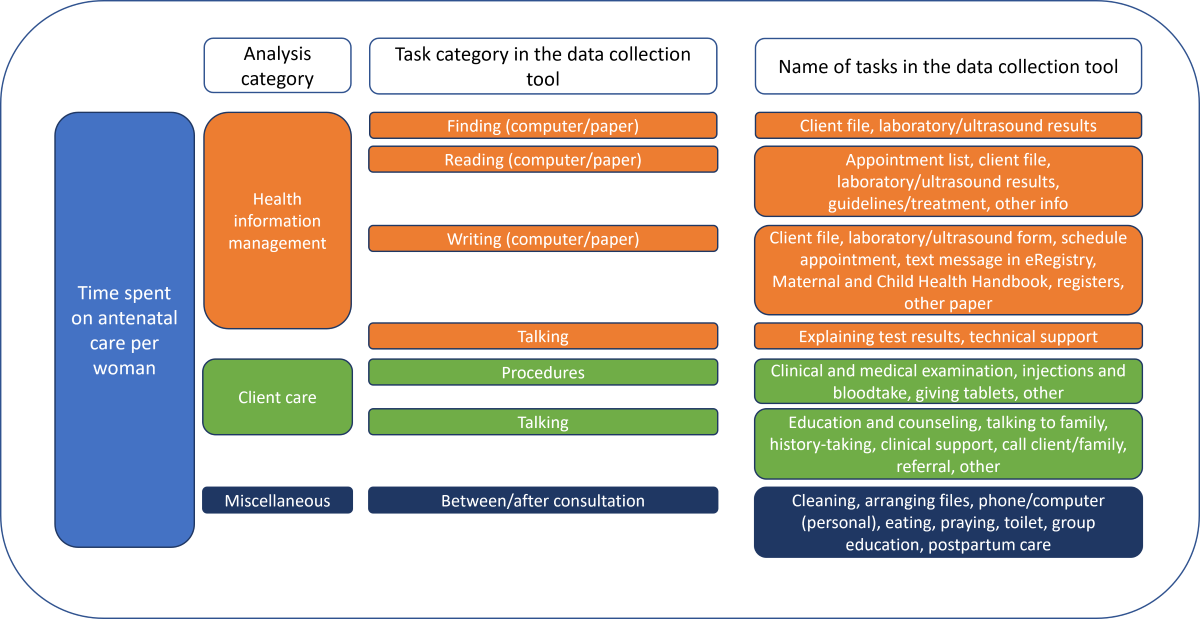
Analysis categories that constitute an antenatal care consultation, task category in the data collection, and name of the task in the data collection tool against each category.

We categorized the tasks in the data collection tool into one of the following six activity types: finding, reading, writing, client care, talking, and miscellaneous [19]. Each antenatal care consultation consisted of differing compositions of the activity types (Figure 1). We applied the task definitions to the final data set and classified each task under one of the following analysis categories: health information management, client care, or miscellaneous (Figure 1). The primary outcome was the time spent on health information management per consultation, defined as the time spent per client consultation on all tasks involving “finding,” “reading,” “writing,” and some tasks listed under “talking” (Figure 1). Time spent on documentation in registers after consultation hours was averaged across the client consultations and added to the health information management time.

In addition to time-motion data, we collected a predefined set of characteristics of clinics and care providers included in the study. No identifiable data were collected on care providers or clients.

It was not possible to blind data collectors, care providers, or clients with respect to allocation owing to the nature of the intervention (eRegistry versus paper-based documentation). However, it was possible to blind them with respect to outcome measurement; that is, data collectors, care providers, and clients were not informed of what was being measured. To reduce possible bias due to lack of blinding to allocation, data collectors were instructed to observe full working days and record data beyond that needed for the computation of the study outcomes. The statistician (CJR) was not involved in data collection and was blinded to treatment allocation for the analyses of relative differences in time used on health information management (primary outcome), client consultation, and client care. It was not possible to blind the statistician to treatment allocation for the analyses of time used finding, reading, and writing files because the treatment allocation was obvious (care providers in the control group could not use a computer for these tasks).

### Statistical Analysis

We calculated the sample size by assuming that clinics in the control group (paper-based documentation) spend an average of 10 minutes on health information management per client and that time use would vary more in the intervention (SD 5 minutes) than control clinics (SD 2 minutes). We calculated that a minimum sample size of 8 observations per clinic from each of the 24 clinics would be needed to detect a 25% difference (judged to be clinically meaningful) with 90% power and 5% significance.

We checked data from pilot observations prior to the study for mean (SD) values of time spent on each consultation, health information management, and client care for observations as recorded by each of the observers. We calculated interrater reliability using Cohen κ [20] for the total number of task categories per consultation recorded by each pair of observers.

We present sample means for total consultation time, health information management time, and time spent on client care in the control and intervention groups. We transformed time use to the logarithmic scale and used mixed-effects linear regression to estimate relative time use. Use of the logarithmic scale facilitates estimation of relative time and addresses the issue that time use is a nonnegative quantity that is often positively skewed (ie, many consultations are of “typical” duration, but some are much longer). We adjusted for the variables used to stratify [21] and constrain randomization (cluster size and lab availability) [22], which we modeled as fixed effects. We reasoned that booking visits (new pregnancy registrations) are fundamentally different to follow-up antenatal care visits (we anticipated that they would be of longer duration); therefore, we also adjusted for visit type as a fixed effect. We used random intercepts at the level of clinic to model the cluster-randomized design and used clustered sandwich estimation to account for possible within-observer clustering. We exponentiated to obtain estimates of relative time use. To aid interpretation of the estimated quantities, we computed marginal mean times used in total and on health information management, client care, finding, reading, and writing, with respect to cluster size, laboratory availability, and visit.

We followed the intention-to-treat principle for all analyses: clusters (and hence participants) were analyzed in the arms to which they were randomized, and all clusters and participants were included in the analyses. No data were missing. Sample size calculations and statistical analyses were performed using Stata 16 (StataCorp LLC). Protocol deviations are documented in Multimedia Appendix 1.

### Ethics Approval

Approvals for conducting the eRegTime study were obtained from the Palestinian Health Research Council (PHRC/HC/208/17) and the Regional Committee for Medical and Health Research Ethics in Norway (2017/400), and from the Ministry of Health, Palestine. Participating clinics and care providers were informed of the data collection. Owing to the inherent hesitancy of clients in the study area in placing signatures on documents, verbal informed consent was obtained from all clients prior to start of observation of antenatal care in the study clinics. This study is reported in accordance with the CONSORT-EHEALTH (Consolidated Standards of Reporting Trials of Electronic and Mobile HEalth Applications and onLine TeleHealth) guidelines (Multimedia Appendix 2).

## Results

During the pilot data collection prior to the study, observers 1 and 3 recorded slightly different mean times spent on a consultation (18.3, SD 2.2 minutes vs 17.5, SD 1.3 minutes), on health information management (9.9, SD 2.8 minutes vs 8.9, SD 2.4 minutes), and on client care (8.3, SD 2.3 minutes vs 8.3, SD 1.8 minutes). Interrater reliability (Cohen κ) for the total number of task categories per consultation was 0.67, indicating substantial agreement [23]. Recordings of observers 2 and 4 were closer in terms of mean times spent on consultation (11.5, SD 7.9 minutes vs 11.2, SD 7.7 minutes), on health information management (5.7, SD 3.7 minutes vs 5.1, SD 3.1 minutes), and on client care (5.4, SD 4.5 minutes vs 5.8, SD 5.0 minutes). Interrater reliability (Cohen κ) for the total number of task categories per consultation was 0.78, indicating substantial agreement as before.

**Figure 2:**
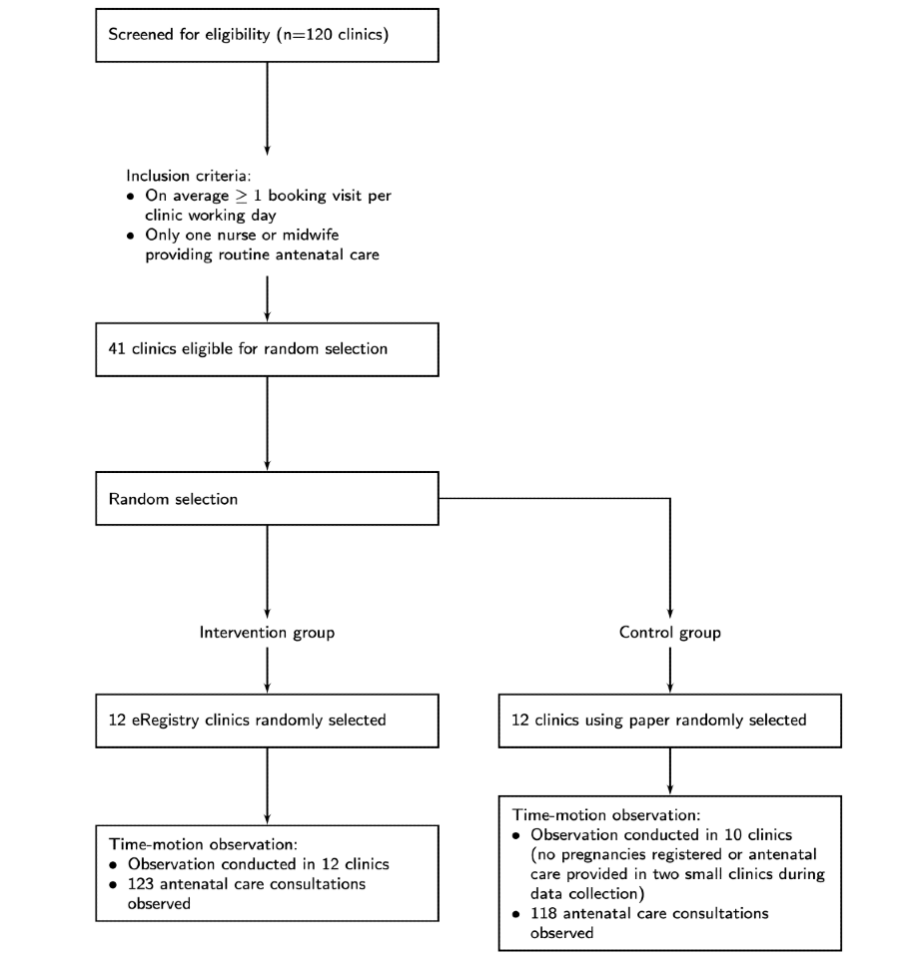
Participant flow diagram.

From August to December 2018, data collection was completed at 10 control clinics and 12 intervention clinics, corresponding to a total observation time of 66 hours 26 minutes, and 61 hours 17 minutes, respectively. Four clinics in the control group and 2 in the intervention group were observed for 2 working days, while the remaining were observed for 1 working day. Data could not be collected from 2 control clinics, since they were small clinics that neither registered new pregnancies nor provided follow-up antenatal care during the data collection period. A total of 118 antenatal care consultations were observed in the control group, of which 19 were booking visits, while 123 antenatal care consultations were observed in the intervention group, of which 11 were booking visits (new pregnancy registrations) (Figure 2).

The age and years of experience of nurses and midwives providing routine antenatal care were comparable across the control and intervention groups (Table 1). A laboratory was available in 6 control clinics and 7 intervention clinics.

**Table 1:** Characteristics of clinics and care providers included in the eRegTime study.

Characteristics	Control group, mean (SD)	Intervention group, mean (SD)
Age (years) of the care provider^a^	42.6 (9.3)	43.1 (8.7)
Care provider’s years of experience	16.0 (8.1)	17.4 (8.8)
New pregnancy registrations per month during the data collection period	5.5 (2.2)	5.8 (4.9)
Days of service provision per week	1.7 (1.3)	2.0 (1.5)

^a^Nurse or midwife providing routine antenatal care in primary care clinics.

Table 2 presents comparisons of control and intervention groups of total time spent per consultation, on health information management (primary outcome) and on client care, for booking visits (new pregnancy registrations), follow-up antenatal care visits, and overall. Total consultation time was shorter in the intervention group (sample mean 11.99 minutes) than the control group (sample mean 15.56 minutes). The intervention group spent 74% (adjusted relative time 0.74, 95% CI 0.60-0.90; *P*=.003) of the time spent per client consultation in the control group. The intervention appears to have reduced consultation times of follow-up antenatal care visits (adjusted relative time 0.72, 95% CI 0.58-0.90; *P*=.004), but not booking visits (Table 2). Health information management time per client consultation was shorter in the intervention group (sample mean 6.64 minutes) than in the control group (sample mean 9.84 minutes), with an adjusted relative time of 0.70 (95% CI 0.59-0.82; *P*<.001). Client care was not different in the intervention group compared to that in the control group overall (sample mean 5.01 minutes vs 4.91 minutes; adjusted relative time 0.85, 95% CI 0.64-1.13), or for booking or follow-up visits (Table 2).

**Table 2:** Analysis of total time use and time use on health information management and client care in the intervention and control groups.

		Sample means (minutes)^a^			Relative time (intervention/control)			
		Control	Intervention	Sample (unadjusted)		Adjusted^b^	95% CI^c^	*P* value^c^
**Total time^d^**								
	Any visit	15.56	11.99	0.77		0.74	0.60-0.90	.003
	Booking^e^	29.36	24.80	0.84		0.96	0.66-1.39	.82
	Follow-up	12.91	10.68	0.83		0.72	0.58-0.90	.004
**Health information management**								
	Any visit	9.84	6.64	0.67		0.70	0.59-0.82	<.001
	Booking	18.91	15.26	0.81		0.96	0.61-1.50	.85
	Follow-up	8.10	5.72	0.71		0.69	0.60-0.79	<.001
**Client care**								
	Any visit	4.91	5.01	1.02		0.85	0.64-1.13	.27
	Booking	8.56	8.82	1.03		0.79	0.36-1.72	.55
	Follow-up	4.22	4.66	1.10		0.84	0.60-1.19	.33

^a^Sample means were not computed on the log scale.

^b^Estimates of relative time use were adjusted for the stratification variable, cluster size, lab availability, and booking visit.

^c^95% CIs and *P* values were adjusted for possible cluster effects due to the cluster-randomized controlled trial design and observer.

^d^Total time includes activities not accounted for in health information management and client care.

^e^Booking refers to a new pregnancy registration.

Table 3 shows the relative differences in time used on activities such as finding, reading, and writing—the main activity types that constitute health information management per client consultation—in the intervention versus control groups. The mean time spent on finding files and laboratory test results was longer in the intervention than in the control group (sample means 0.92 vs 0.68 minutes; adjusted relative time 1.30, 95% CI 1.16-1.45) (Table 3). On the other hand, we estimated that the intervention group used only 68% of the time as the control group on writing tasks overall (adjusted relative time 0.68, 95% CI 0.56-0.83) and for follow-up antenatal care visits (adjusted relative time 0.65, 95% CI 0.59-0.72).

**Table 3:** Analysis of time used in finding, reading, and writing (components of health information management) in the intervention and control groups.

			Sample means (minutes)^a^					Relative time (intervention/control)						
			Control		Intervention		Sample (unadjusted)			Adjusted^b^		95% CI^c^		*P* value^c^
**Finding**														
	Any visit	0.68		0.92		1.34			1.30		1.16-1.45		<.001	
	Booking^d^	1.21		1.45		1.20			1.96		0.66-5.79		.22	
	Follow-up	0.60		0.86		1.42			1.22		1.09-1.36		<.001	
**Reading**														
	Any visit	1.20		0.72		0.60			0.92		0.73-1.15		.47	
	Booking	2.10		0.39		0.19			0.80		0.65-0.98		.03	
	Follow-up	0.99		0.73		0.74			0.92		0.69-1.22		.57	
**Writing**														
	Any visit	8.48		5.50		0.65			0.68		0.56-0.83		<.001	
	Booking	16.48		13.73		0.83			1.30		0.55-3.05		.55	
	Follow-up	6.94		4.61		0.66			0.65		0.59-0.72		<.001	

^a^Sample means were not computed on the log scale.

^b^Estimates of relative time use were adjusted for the stratification variable, cluster size, lab availability, and booking visit.

^c^95% CIs and *P* values were adjusted for possible cluster effects due to the cluster-randomized controlled trial design and observer.

^d^Booking refers to a new pregnancy registration.

## Discussion

### Principal Findings

We conducted a continuous observation time-motion study in the setting of a cluster-randomized controlled trial. The intervention, a digital maternal and child health registry (eRegistry), reduced the time spent on health information management without affecting time spent on client care. More time was saved on health information management for follow-up antenatal care visits compared to booking visits (new pregnancy registrations). Our results suggest that adoption of digital tools like the eRegistry in antenatal clinics in LMICs would be expected to meaningfully reduce time spent by care providers on writing tasks.

### Comparison With Prior Work

The intervention group in our study had significantly shorter consultation times. In contrast, an assessment in primary care clinics using paper versus electronic health records in Jordan did not show a significant difference in consultation times [24]. However, this result is likely explained by inadequate sample size, since consultation times in clinics using paper records showed wide variations. A multicountry study conducted in Tanzania and Ghana showed that a digital clinical decision support system resulted in increased time spent on antenatal care, although a nonsignificant increase in time was also observed in the nonintervention sites [25]. Booking visits (new pregnancy registrations) typically take longer than follow-up antenatal care visits, as also shown by the results of our study. The eRegistry provides longitudinal digital client records of pregnancies [12], and the care provider can review information from all prior antenatal contacts and proceed with only documenting new information. This is likely to have resulted in time saved on health information management during follow-up antenatal care visits in the intervention group. A time-motion study in Uganda conducting an evaluation of the effect of client summaries, equivalent to eRegistry’s longitudinal client records, found a reduction in time spent on client consultation [26].

Of the subcategories constituting health information management, we estimated a reduction in time spent on writing tasks in the intervention group (sample mean 5.50 minutes vs 8.48 minutes; relative time 0.68, 95% CI 0.56-0.83), amounting to substantial time saved for care providers and the health system. Similar to our results, a systematic review of time efficiency of computer- versus paper-based documentation systems showed a reduction in documentation times with the use of electronic health records of up to 25% for nurses using digital point-of-care tools [2]. In our study, nurses in the control group documented the same information in paper-based client records during consultations and in reporting forms after consultation hours. Redundancies in documentations due to this common practice of maintaining parallel paper-based systems in settings with DHIs have proven to be time-consuming for care providers [10,25]. The eRegistry can generate automated aggregate reports based on digital data entry, and the reduction in time spent on health information management may be attributed to the elimination of some of the dual documentations in the intervention group. A similar digital system in Kenya, which replaced dual documentations, also resulted in time saved for health workers [11]. Further elimination of redundant documentation in register books in the intervention clinics can potentially result in more time saved.

Decreased time spent on patient care owing to the use of DHIs such as digital client records is a commonly cited concern among care providers [27,28]. In our study, there was no difference in the time spent on direct patient care between the two groups. Finding client files and test results was the only activity type that the intervention group appeared to spend more time on than the control group, possibly because of the fundamental differences in doing digital versus paper-based searches; client searches in the eRegistry require 2 or more personal identifying information.

### Strengths and Limitations

Time-motion observations, as in our study, have been shown to be more precise in capturing time data as well as less prone to self-report biases compared to other methodologies such as work sampling and self-reporting surveys [29,30]. Since we sampled from a cluster-randomized controlled trial, clinics using paper-based documentations (control group) were likely to be comparable to the eRegistry clinics (intervention group) in all aspects except for the intervention. Time since first implementation is a crucial factor in the evaluation of time efficiency. A systematic review showed that evaluations of electronic health records performed soon after implementation tend to show reduced documentation times compared to those performed later [31]. The eRegTime study was conducted 18 months after the rollout of the eRegistry. We believe that this allowed sufficient time for care providers to acclimatize to the eRegistry such that our estimates reflect differences with respect to routine clinical practice rather than excessively large effects attributable to a new system. Unlike many other LMICs, the West Bank has no vertical, donor-driven programs for maternal and child health, which demand separate reporting from primary health care. A unified system of automated digital public health reports was relatively easy to roll out—a process that might be challenging in many LMICs.

The study has some limitations. First, our inclusion criteria were based on clinic characteristics from 2014. The sample size was slightly smaller than planned since 2 clinics did not have any antenatal care visits during the data collection period. Second, we conducted only one interrater reliability assessment prior to the study and did not conduct any during the data collection period; however, we did adjust for possible within-observer clustering (see *Statistical Analysis* in *Methods* and protocol deviations in Multimedia Appendix 1).

### Conclusions

The eRegistry with clinical decision support and automated reporting results in reduced time spent on health information management, possibly without adversely affecting client care time. DHIs that reduce workloads for care providers are perceived as more acceptable, which is crucial for scaling up and sustainability of implementations.

## Appendix

Multimedia Appendix 1 Protocol deviations.

Multimedia Appendix 2 CONSORT‐EHEALTH checklist (V.1.6.1).

## Abbreviations

CISMAC: Centre for Intervention Science in Maternal and Child Health
DHI: digital health intervention
DHIS2: District Health Information Software 2
LMIC: low- and middle-income countries
WHO: World Health Organization
