# Supplementary material for: eRegTime—Time Spent on Health Information Management in Primary Health Care Clinics Using a Digital Health Registry Versus Paper-Based Documentation: Cluster-Randomized Controlled Trial
Source: JMIR Form Res. 2022 May 13;6(5):e34021. doi: 10.2196/34021 (PMC9143771; doi:10.2196/34021)
Supplement: Multimedia Appendix 1 [file formative_v6i5e34021_app1.docx]

# Multimedia appendix – Protocol Deviations

We did not plan to transform to the log scale to estimate relative time use, however prior to analysis we judged that in addition to the statistical rationale (i.e., addressing non-negativity and skew) the intervention was likely to change all aspects of managing health information and would therefore be better modelled as a multiplicative effect (i.e., additive on the logarithmic scale). We did not plan to adjust for clustering within observer but chose to do so prior to analysis because it is plausible that systematic differences may exist between observers. This is further justified by inter-rater reliability, which was relatively high, but not perfect. We did not plan in our protocol how to analyze time used finding, reading, or writing files; these secondary outcomes were planned after data were available.
